# Supplementary material for: Identification of photoperiod-induced specific miRNAs in the adrenal glands of Sunite sheep (Ovis aries)
Source: Front Vet Sci. 2022 Jul 22;9:888207. doi: 10.3389/fvets.2022.888207 (PMC9354845; doi:10.3389/fvets.2022.888207)
Supplement: Supplementary file 1 [file Data_Sheet_1.ZIP › Supplementary file/miRDeep2 file.zip!/miRDeep2 file/Know_Structure/LP42A2/oar-mir-30b.pdf]

miRBase precursor : oar-mir-30b
Total read count : 35574
oar-miR-30b read count : 35338
remaining reads : 236

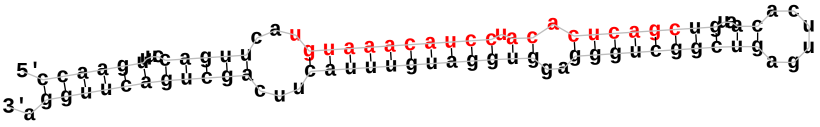

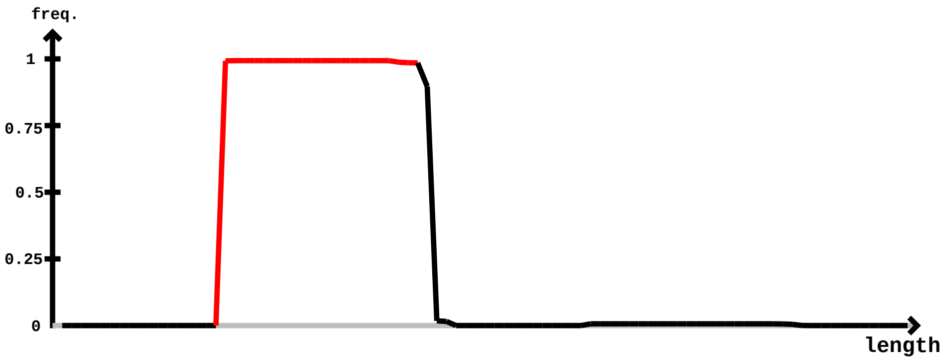


```
                      oar-miR-30b
5'- ccaaguuuccaguucauguaaacauccuacacucagcuguaacacuugagucggcugggagguggauguuuacuucagcugacuugga   -3'    exp
    ((((((...(((((...((((((((((.((.((((((((..((......))))))))))..))))))))))))...))))))))))).   reads   mm   sample
    ...............auguaaacauccuacacucagcu..................................................   1       0    seq
    ................uguaaacauccuacacu.......................................................   4       0    seq
    ................uguaaacauccCacacuc......................................................   2       1    seq
    ................uguaaacauccuacacuc......................................................   133     0    seq
    ................uguGaacauccuacacuc......................................................   1       1    seq
    ................uguaaacauccuacacuU......................................................   61      1    seq
    ................uguaaacauccuacacuca.....................................................   80      0    seq
    ................uguaaacauccuacacucag....................................................   16      0    seq
    ................uguaaacauccuacacucagc...................................................   3141    0    seq
    ................uguaaacauccuacacucagcu..................................................   31243   0    seq
    ................uguaaacauccuacacucagcug.................................................   78      0    seq
    ................uguaaacauccuacacucagcugu................................................   550     0    seq
    ................uguaaacauccuacacucagcugua...............................................   3       0    seq
    .................guaaacauccuacacucagcu..................................................   24      0    seq
    ..................uaaacauccuacacucagcu..................................................   1       0    seq
    ...............................................gagucggcugggagguggauguuuacuuc............   1       0    seq
    ......................................................cugggagguggauguuua................   1       0    seq
    ......................................................cugggagguggauguuuac...............   2       0    seq
    ......................................................cugggagguggauguuuacu..............   21      0    seq
    ......................................................cugggagguggauguuuacuu.............   49      0    seq
    ......................................................cugggagguggauguuuacuuc............   143     0    seq
    ......................................................cugggagguggauguuuacuuca...........   13      0    seq
    .......................................................ugggagguggauguuuacuu.............   1       0    seq
    .......................................................ugggagguggauguuuacuuc............   2       0    seq
    .......................................................ugggagguggauguuuacuuca...........   3       0    seq
```
